# Structural insight into an anti-BRIL Fab as a G-protein-coupled receptor crystallization chaperone

**DOI:** 10.1107/S205979832300311X

**Published:** 2023-04-26

**Authors:** Hikaru Miyagi, Michihiko Suzuki, Mai Yasunaga, Hidetsugu Asada, So Iwata, Jun-ichi Saito

**Affiliations:** a R&D Division, Kyowa Kirin Co. Ltd, Tokyo, Japan; b R&D Division, Kyowa Kirin Co. Ltd, Shizuoka, Japan; c CMC R&D Center, Kyowa Kirin Co. Ltd, Shizuoka, Japan; d Department of Cell Biology, Graduate School of Medicine, Kyoto University, Kyoto, Japan; e RIKEN, SPring-8 Center, Hyogo, Japan; UNSW Sydney, Australia

**Keywords:** crystal structure, crystal packing, apocytochrome *b*_562_, GPCRs, crystallization chaperones, anti-BRIL Fabs

## Abstract

Structure determination of G-protein-coupled receptors (GPCRs) is key for the successful development of efficient drugs targeting GPCRs. BRIL is a thermostabilized apocytochrome *b*_562_ (with M7W/H102I/R106L mutations) from *Escherichia coli* and is often used as a GPCR fusion protein for expression and crystallization. SRP2070Fab, an anti-BRIL antibody Fab fragment, has been reported to facilitate and enhance the crystallization of BRIL-fused GPCRs as a crystallization chaperone. This study was conducted to characterize the high-resolution crystal structure of the BRIL–SRP2070Fab complex. The structure of the BRIL–SRP2070Fab complex was determined at 2.1 Å resolution. This high-resolution structure elucidates the binding interaction between BRIL and SRP2070Fab. When binding to BRIL, SRP2070Fab recognizes conformational epitopes, not linear epitopes, on the surface of BRIL helices III and IV, thereby binding perpendicularly to the helices, which indicates stable binding. Additionally, the packing contacts of the BRIL–SRP2070Fab co-crystal are largely due to SRP2070Fab rather than BRIL. The accumulation of SRP2070Fab molecules by stacking is remarkable and is consistent with the finding that stacking of SRP2070Fab is predominant in known crystal structures of BRIL-fused GPCRs complexed with SRP2070Fab. These findings clarified the mechanism of SRP2070Fab as a crystallization chaperone. Moreover, these data will be useful in the structure-based drug design of membrane-protein drug targets.

## Abstract 2

The structure of a complex between BRIL and an anti-BRIL antibody (SRP2070Fab) has been determined at high resolution. This work presents a detailed elucidation of the interaction between BRIL and SRP2070Fab, which may help to improve the function of SRP2070Fab as a crystallization chaperone for membrane proteins.

## 1. Introduction

G-protein-coupled receptors (GPCRs) are the largest family of membrane proteins and regulate many physiological functions associated with the nervous and cardiovascular systems, metabolism and inflammation, among others (Venkata­krishnan *et al.*, 2013 ▸). Therefore, GPCRs have been an important target in drug development. Since the 1990s, structure elucidation of the drug candidate–target protein complex has widely been used to optimize hit compounds to lead compounds by rational design based on the obtained structural information, an approach called structure-based drug design (SBDD; Lee *et al.*, 2018 ▸; Shoichet & Kobilka, 2012 ▸). Although structure determination is key for the success of SBDD, technical difficulties continue to persist, especially for membrane-protein targets involved in protein production, purification or crystallization. SBDD has extensively been applied to GPCRs since 2007, when the first crystal structure of a human GPCR was determined (Cherezov *et al.*, 2007 ▸; Rasmussen *et al.*, 2007 ▸). X-ray crystallography was almost the only available method for the structure determination of GPCRs until the development of single-particle cryo-electron microscopy (cryo-EM) analysis in 2017; recently, an increasing number of structures have been determined by cryo-EM analysis (García-Nafría & Tate, 2021 ▸).

Several inactive-state GPCR structures bound to antagon­ists or inverse agonists have been determined by X-ray crystallography, whereas most active-state GPCR structures coupled to heterotrimeric G proteins have been determined by cryo-EM analysis (Congreve *et al.*, 2020 ▸). This tendency is attributed to the range of tractable particle sizes using current cryo-EM technology (García-Nafría & Tate, 2021 ▸). Therefore, while the number of GPCR structures determined by cryo-EM analysis is expected to continue to increase, X-ray crystallo­graphy will remain the method of choice for elucidating GPCR structures below 50 kDa. Another advantage of X-ray crystallography for SBDD is the high throughput of complex structure determination with numerous ligands. SBDD is generally performed in the optimization stage of candidate compounds, with several compounds being screened in the analysis. A series of candidate compound structures bound to a target GPCR can quickly be determined by utilizing the soaking method, in which the compounds are soaked into apoprotein crystals of the GPCR; hence, X-ray crystallo­graphy can be used to accelerate SBDD for GPCRs (Rucktooa *et al.*, 2018 ▸). Thus, X-ray crystallography and cryo-EM serve as complementary methods in assessing the structure–function relationships of GPCRs and for use in SBDD.

BRIL, an engineered four-helix bundle protein, is a thermo­stabilized apocytochrome *b*_562_ protein from *Escherichia coli* that is derived from cytochrome *b*_562_ and stabilized as an apoprotein without the cofactor heme by introducing M7W/H102I/R106L mutations (Chu *et al.*, 2002 ▸). BRIL has been widely used as a GPCR fusion protein since 2012. Structural flexibility can be reduced and thermostability can be improved by replacing the flexible loop of GPCRs with BRIL, which is often necessary for expression and crystallization (Chun *et al.*, 2012 ▸; Xiang *et al.*, 2016 ▸). Several optimizations are required to obtain GPCR constructs suitable for crystallization: (i) the deletion of N-terminal and/or C-terminal domains (Hollenstein *et al.*, 2013 ▸; Jaakola *et al.*, 2008 ▸; Huang *et al.*, 2013 ▸; Warne *et al.*, 2011 ▸), (ii) the implementation of point mutations to improve thermal stability (Vaidehi *et al.*, 2016 ▸) and (iii) the insertion of a fusion protein into the third intracellular loop or into the N-terminus (Xiang *et al.*, 2016 ▸). BRIL is often chosen as a fusion partner, and approximately 20% of reported GPCR structures have been determined as BRIL fusion mutants (Xiang *et al.*, 2016 ▸).

We recently reported an anti-BRIL antibody fragment (SRP2070Fab) that facilitates and enhances the crystallization of BRIL-fused GPCRs and demonstrated that SRP2070Fab can be a versatile crystallization chaperone for BRIL-fused GPCRs (Miyagi *et al.*, 2020 ▸). The structures of two BRIL-fused GPCRs, namely 5HT_1B_ bound to ergotamine and AT_2_R bound to an angiotensin II analog, were determined by complexing the GPCRs with SRP2070Fab. Our study also showed that no undesired structural change occurred on the binding of SRP2070Fab and that this approach can be applied for structural analyses of GPCRs regardless of whether the receptors adopt active or inactive conformations. However, the co-crystal structures of AT_2_R or 5HT_1B_ complexed with SRP2070Fab had low resolution, which was a limitation for characterizing detailed structural features such as the precise interactions within epitopes and paratopes or crystal-packing contacts.

More recently, a series of semi-synthetic anti-BRIL antibodies (sABs) have been reported as tools for the structure determination of BRIL-fused membrane proteins, and the structure of BAG2, an affinity-matured sAB, bound to BRIL was determined at 1.9 Å resolution (Mukherjee *et al.*, 2020 ▸). These studies raise expectations that anti-BRIL antibodies may be promising tools for the structural study of membrane proteins.

In this study, we characterized the high-resolution crystal structure of the BRIL–SRP2070Fab complex, which provides a clearer understanding of the BRIL recognition sites and the crystal-packing interactions. This information clarified the differences between SRP2070Fab and other anti-BRIL antibodies and revealed the advantages of SRP2070Fab as a crystallization chaperone.

## 2. Materials and methods

### 2.1. Expression and purification of BRIL

The amino-acid sequence of BRIL used in the present study was the same as that reported previously (Chu *et al.*, 2002 ▸). The gene encoding a Tobacco etch virus (TEV) protease cleavage site and BRIL (Ala1–Leu106) was synthesized and subcloned into pET-28a(+) using BamHI and HindIII. 6×His-tagged BRIL was expressed in the *E. coli* BL21 (DE3) strain cultured in Luria–Bertani medium. The cultured cells were harvested and lysed after induction with isopropyl β-d-1-thiogalactopyranoside. 6×His-tagged BRIL was first purified by nickel-affinity chromatography. The protein bound to the Ni–NTA agarose resin was eluted with 100 m*M* Tris–HCl pH 7.6, 300 m*M* NaCl, 300 m*M* imidazole, 5 m*M* 3-mercapto-1,2-propanediol. The 6×His tag was cleaved with TEV protease and removed by collecting the flowthrough fraction from the Ni–NTA agarose resin. Further purification was performed by anion-exchange chromatography using a Mono 5/50 GL column (Cytiva) and the peak fractions containing BRIL were collected.

### 2.2. Preparation of SRP2070Fab

SRP2070Fab was prepared according to the method reported previously (Miyagi *et al.*, 2020 ▸). Briefly, hybridomas producing SRP2070 were intraperitoneally administered to MRL/lpr mice. Ascites was collected from the hybridoma-administered mice and SRP2070 was purified by ammonium sulfate precipitation and protein G affinity chromatography. After cleaving SRP2070 with papain, the Fc fragment was removed by collecting the flowthrough fraction from the protein A column. SRP2070Fab was further purified by size-exclusion chromatography (SEC) on a Superdex 200 10/300 GL column equilibrated with phosphate-buffered saline.

### 2.3. Purification of BRIL–SRP2070Fab

BRIL and SRP2070Fab were mixed in a 1:1.2 molar ratio for 1 h on ice before further purification of the complex by SEC using a Superdex 200 Increase 10/300 GL column equilibrated with SEC buffer consisting of 20 m*M* Tris–HCl pH 7.6, 2 m*M* 3-mercapto-1,2-propanediol. Concentrated BRIL–SRP2070Fab (∼20 mg ml^−1^) was used in crystallization experiments.

### 2.4. Crystallization and structure determination of BRIL–SRP2070Fab

Initial crystallization trials were performed by the sitting-drop vapor-diffusion method using a Mosquito crystallization robot (SPT Labtech). All drops were formed by mixing 100 nl protein solution with an equal volume of reservoir solution in 96-well plates, and the drops were equilibrated against 50 µl reservoir solution. Initial crystallization screening using JCSG+ (Molecular Dimensions), ProPlex (Molecular Dimensions) and The PEGs II Suite (Qiagen) revealed several promising hit conditions. Crystals of the BRIL–SRP2070Fab complex were finally obtained using a condition composed of 18%(*w*/*v*) polyethylene glycol (PEG) 6000, 100 m*M* Tris–HCl pH 8.5 at 4°C (Supplementary Fig. S1 and Table S1), which was optimized from a hit from the ProPlex screen. The crystals were cryoprotected by soaking them in a solution containing 20%(*v*/*v*) glycerol before flash-cooling in liquid nitrogen.

Diffraction data for the BRIL–SRP2070Fab co-crystal were collected on the X10SA beamline at the Swiss Light Source (SLS) and were processed using *XDS* (Kabsch, 2010 ▸) and *AIMLESS* (Evans & Murshudov, 2013 ▸). The initial phase was determined by molecular replacement using *MOLREP* (Vagin & Teplyakov, 2010 ▸) and *Phaser* (McCoy *et al.*, 2007 ▸). The structures of BRIL (PDB entry 1m6t; Chu *et al.*, 2002 ▸) and of Fab derived from mouse IgG1 (PDB entry 1f58; Stanfield *et al.*, 1999 ▸) were used as search models. The initial structural model was refined by rigid-body and restrained refinement with *REFMAC*5 (Murshudov *et al.*, 2011 ▸). The model of the structure was repeatedly corrected using *Coot* to be consistent in electron density (Emsley *et al.*, 2010 ▸). The interfaces between BRIL and SRP2070Fab and within the crystal packing were analyzed with *PISA* (Krissinel & Henrick, 2007 ▸) and *Molecular Operating Environment* (*MOE*, version 2020.0901; Chemical Computing Group ULC). The buried surface area (BSA) on complex formation was calculated by *PISA* according to the equation BSA_*AB*_ = (ASA_*A*_ + ASA_*B*_ − ASA_*AB*_)/2, where BSA_*XY*_ is the buried surface area when molecules *X* and *Y* form a complex and ASA_*X*_ is the total accessible surface area of molecule *X*. Structural figures were prepared using the *CCP*4*mg* molecular-graphics software (Potterton *et al.*, 2002 ▸) and *PyMOL* version 0.99 (DeLano, 2002 ▸).

## 3. Results and discussion

A complete data set at 2.1 Å resolution was obtained from the BRIL–SRP2070Fab co-crystal, which belonged to space group *C*2 with unit-cell parameters *a* = 93.83, *b* = 75.24, *c* = 152.23 Å, β = 99.67° and contained two BRIL–SRP2070Fab complexes in the asymmetric unit. The crystallographic statistics of data collection and refinement are summarized in Table 1 ▸.

**Figure 1:**
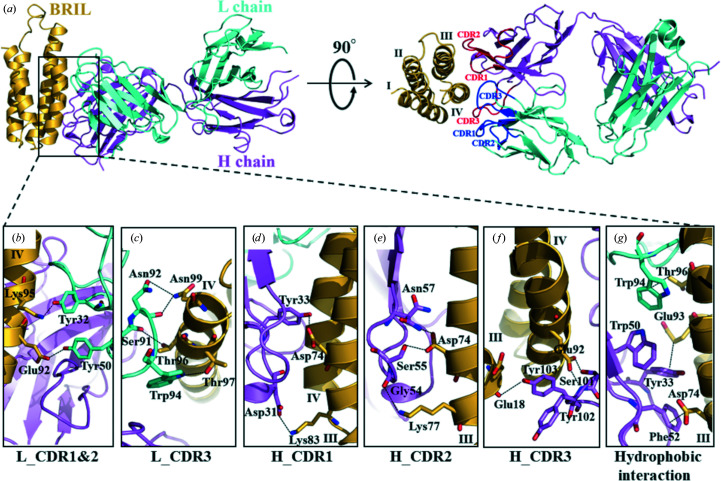
Structure of the BRIL–SRP2070Fab complex and the interaction between BRIL and SRP2070Fab. (*a*) Overall structure of the BRIL–SRP2070Fab complex. The protein structures are represented as ribbon models. BRIL is indicated in ocher, heavy (H) chain is indicated in purple and light (L) chain is indicated in light blue. (*b*)–(*f*) Interactions between BRIL and complementarity-determining regions (CDRs) are shown. The interacting side chains are shown as sticks, and hydrogen bonds and salt bridges are shown as dashed lines. (*e*) Asn57^H_CDR2^ adopts a double conformer. (*g*) Hydrophobic interactions between BRIL and SRP2070Fab are shown.

The overall structure of the co-crystal showed that SRP2070Fab binds to BRIL by recognizing conformational epitopes, not linear epitopes, of the natively structured BRIL. All six complementarity-determining regions (CDRs) in the heavy (H) and light (L) chains cooperatively enable the recognition of BRIL, and CDR1 and CDR3 of the H chain that pinch helix IV of BRIL from both sides are remarkable compared with the others (Fig. 1 ▸ *a*, right). Conformational epitopes, *i.e.* residues within 4 Å of SRP2070Fab, are extensively spread over helices III and IV of BRIL (Figs. 1 ▸ *b*–1*f*). The BSA on antigen–antibody complex formation is 923.2 Å^2^ on average for the two BRIL–SRP2070Fab complexes in the asymmetric unit (Supplementary Table S2*a* ), which is larger than the average for known antigen–antibody interfaces of 534 ± 157 Å (Reis *et al.*, 2022 ▸). Hydrophilic interactions such as hydrogen bonds and salt bridges play major roles in BRIL recognition: seven hydrogen bonds and one salt bridge are formed between the H chain and BRIL. One hydrogen bond and one salt bridge are found in CDR1, and three hydrogen bonds are found in both CDR2 and CDR3. Six hydrogen bonds are also formed between the L chain and BRIL. One hydrogen bond is formed in CDR1 and CDR2, and four hydrogen bonds are formed in CDR3. The detailed inter­actions are described in Supplementary Table S3. Although hydrophobic interactions contribute less to BRIL recognition, it is noteworthy that a sequence of aromatic residues, Trp94^L_CDR3^, Tyr33^H_CDR1^, Trp50^H_CDR2^ and Phe52^H_CDR2^, provide aromatic interactions with BRIL helices III and IV (Fig. 1 ▸ *g*). Considering the number and the distance of the interactions, Asp74 and Glu92 are considered to be at the center of the epitope (Fig. 1 ▸, Supplementary Table S3). The high-resolution structure revealed that both the epitopes and the paratopes are conformationally and spatially extended, and thus SRP2070Fab recognizes BRIL on the surface but not at a particular spot or at a linear amino-acid sequence. It is noteworthy that all six CDRs cooperatively bind to BRIL, which indicates the stability of the BRIL–SRP2070Fab complex; therefore, this aspect of SRP2070Fab may be advantageous for it to serve as a crystallization chaperone (Fig. 1 ▸, Supplementary Table S3).

**Figure 2:**
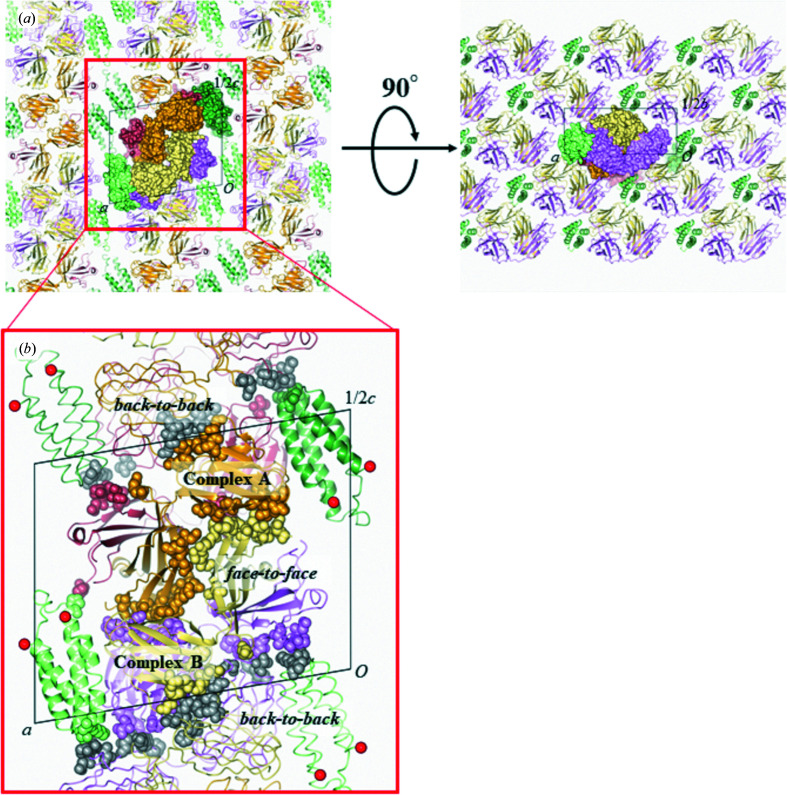
Molecular packing of the BRIL–SRP2070Fab co-crystal. (*a*) Molecular packing of the BRIL–SRP2070Fab crystal along the *ac* plane and the *ab* plane. An asymmetric unit is denoted as a rectangle drawn in black lines. Two BRIL–SRP2070Fab complexes (complexes *A* and *B*) in the asymmetric unit are shown as a surface, while molecules outside the asymmetric unit are shown as ribbon models. BRIL, SRP2070Fab heavy chain and SRP2070Fab light chain are shown in green, dark red and orange for complex *A* and in light green, pink and khaki for complex *B*, respectively. (*b*) Close-up image of the stacking along the *c* axis of the BRIL–SRP2070Fab crystal. Residues making contacts with neighboring complexes are depicted as spheres. Residues from neighbors outside of the asymmetric unit are shown in gray for better distinction. The N- and C-termini of BRIL are marked by red spheres.

**Figure 3:**
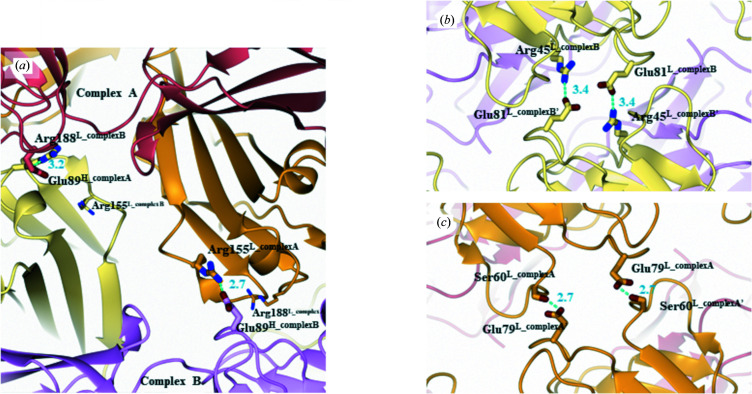
Crystal-packing interactions in the BRIL–SRP2070Fab co-crystal. Interactions observed within (*a*) face-to-face stacking between complexes *A* and *B*, (*b*) back-to-back stacking between complex *A* and its symmetry mate *A*′ and (*c*) back-to-back stacking between complex *B* and its symmetry mate *B*′. SRP2070Fab molecules are represented by ribbon models. The heavy and light chains of SRP2070Fab are shown in dark red and orange for complex *A* and in pink and khaki for complex *B*, respectively. Residues involved in the interactions are depicted as stick models. Salt-bridge or hydrogen-bond interactions are indicated as cyan dashed lines.

To understand the properties of SRP2070Fab with regard to the crystallization of BRIL-fused membrane proteins, we explored the crystal packing of the high-resolution structure of the BRIL–SRP2070Fab complex. SRP2070Fab molecules were found to be tightly packed in the crystal, making contacts and interacting with each other. The crystal packing of the BRIL–SRP2070Fab co-complex is largely due to SRP2070Fab rather than to BRIL; that is, SRP2070Fab contributes almost 80% of the BSA due to crystal packing (Supplementary Table S2*b* ). The results of the crystal-packing analysis showed that several BRIL–SRP2070Fab complexes stack against each other along the *c* axis in a face-to-face or back-to-back manner, while the complex molecules are ordered by side-by-side contacts within the *ab* plane (Fig. 2 ▸ *a*). Here, the side exposing the N- and C-termini of BRIL is defined as the ‘face’ of SRP2070Fab; that is, the face of SRP2070Fab is exposed to the receptor during fusion with BRIL (Supplementary Fig. S2). Stacking along the *c* axis is important because it enables perpendicular interactions with the layer of lipid or detergent mimicking the cellular membrane, which is necessary for the three-dimensional growth of membrane-protein crystals (Fig. 2 ▸ *b*). Face-to-face stacking is predominant among all of the crystal contacts contributed by SRP2070Fab. Two BRIL–SRP2070Fab complexes facing each other are the components of an asymmetric unit; thus, they are not crystallographically equivalent and are hereafter referred to as complexes *A* and *B* (Fig. 2 ▸ *b*). Two remarkable salt bridges, namely Glu89^H_complex*A*^–Arg188^L_complex*B*^ and Glu89^H_complex*B*^–Arg155^L_complex*A*^, are found in the face-to-face stacking (Fig. 3 ▸ *a*). Although Glu89^H^ is involved in both salt bridges, the basic counter-residues are not conserved; this is because complexes *A* and *B* are nearly, but not perfectly, in twofold symmetry. Both of the other back-to-back stacking contacts along the *c* axis have the crystallo­graphic twofold symmetries between complexes *A* and *A* or between complexes *B* and *B*. The BSA for *B*–*B* stacking is more than twice larger than that from *A*–*A* stacking; therefore, *B*–*B* stacking is more dominant in crystal packing than *A*–*A* stacking (Fig. 2 ▸ *b*). *B*–*B* stacking is stabilized by symmetric salt bridges between Arg45^L_complex*B*^ and Glu81^L_complex*B*^, while only weaker hydrogen bonds were observed between Ser60^L_complex*A*^ and Glu79^L_complex*A*^ at the *A*–*A* stacking boundary (Figs. 3 ▸ *b* and 3 ▸ *c*). The stacking interactions are summarized in Supplementary Table S2(*c*).

In our previous study, crystal structures of two BRIL-fused GPCRs, 5HT_1B_-BRIL and AT_2_R-BRIL, were determined in complex with SRP2070Fab (Miyagi *et al.*, 2020 ▸). As SRP2070Fab provides the dominant crystal packing in both crystal structures, we concluded that SRP2070Fab is a promising crystallization chaperone for BRIL-fused GPCRs. Although the packing interactions in this study are not exactly reproduced in the structures of SRP2070Fab complexed with BRIL-fused GPCRs, the conceptual principle of crystallization assistance by SRP2070Fab is commonly based on face-to-face, back-to-back or face-to-back stacking of SRP2070Fab (Supplementary Figs. S3*a* and S3*b* ).

**Figure 4:**
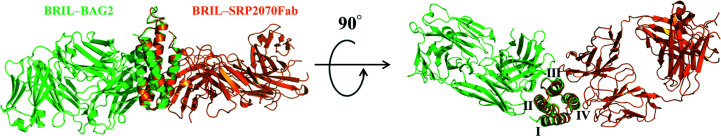
Comparison of two anti-BRIL antibodies (SRP2070Fab and BAG2). The structures of BRIL–SRP2070Fab and BRIL–BAG2 (PDB entry 6cbv) are superimposed. The structures are shown in two directions. BRIL–SRP2070Fab is indicated in ocher and BRIL–BAG2 is indicated in green.

BAG2, another anti-BRIL Fab fragment, is also useful for characterizing the structural biology of membrane proteins (Mukherjee *et al.*, 2020 ▸). Structural comparisons between SRP2070Fab and BAG2 (PDB entry 6cbv) complexed with BRIL clearly show a difference in the extent of BRIL recognition by the Fab fragments. Although both Fab fragments recognize conformational epitopes that are three-dimensionally spread on the BRIL surface and bind nearly at right angles to the BRIL helices, they bind to BRIL from quite different directions: SRP2070Fab recognizes helices III and IV of BRIL in the manner described above, while BAG2 recognizes helices II and III from the opposite side of BRIL (Fig. 4 ▸). Another remarkable difference is that the face of SRP2070Fab is perpendicular to BRIL, while that of BAG2 is coplanar with the BRIL helices, which would impact the crystal growth of BRIL-fused membrane proteins, assuming that the stacking of SRP2070Fab facilitates crystal growth in a direction perpendicular to the hydrophobic membrane layer. In fact, the packing of the BRIL–BAG2 co-crystal is more complex than that of the SRP2070Fab–BRIL co-crystal, and no significant stacking can be found in the Fab portion of the BRIL–BAG2 co-crystal structure (Supplementary Fig. S4).

We then determined the BRIL–SRP2070Fab co-crystal structure at a high resolution that was sufficient to elucidate the detailed structural features of BRIL recognition and the crystal-packing interactions.

Smaller antibody fragments could be another candidate as crystallization chaperones owing to the highly compact crystal packing. ScFv (single-chain Fv) or Fv-clasp, which are new technologies for generating stable Fv fragments (Arimori *et al.*, 2017 ▸), may be applicable to SRP2070.

Additionally, the high-resolution structure of the BRIL–SRP2070Fab complex can be used as a fine search model for complex structures of BRIL-fused membrane proteins and SRP2070Fab. This could be a crystallographic benefit to improve the phase given by molecular replacement in cases where the initial phase determined solely from the membrane-protein portion is not adequate. The high-resolution BRIL–SRP2070Fab co-crystal structure is also expected to be a good starting point for structure building in cryo-EM structure determination.

We believe that our findings will provide an impetus for the structure determination of BRIL-fused membrane proteins complexed with SRP2070Fab and facilitate the SBDD of membrane-protein drug targets.

## 4. Conclusions

In this study, we determined the high-resolution structure of the BRIL–SRP2070Fab complex. This structure enabled us to elucidate the interactions between BRIL and SRP2070Fab. All six CDRs in the heavy and light chains cooperatively enable the recognition of BRIL. This result suggests a strong binding affinity between BRIL and SRP2070Fab. Additionally, detailed information on the crystal packing was clarified by this structure. This result confirms that SRP2070Fab promotes crystallization packing, as suggested in a previous report (Miyagi *et al.*, 2020 ▸), indicating that SRP2070Fab is useful as a crystallization chaperone for BRIL fusion proteins.

## Supplementary Material

PDB reference: BRIL–SRP2070Fab complex, 7xrz

Supplementary Tables and Figures. DOI: 10.1107/S205979832300311X/nj5317sup1.pdf

## Figures and Tables

**Table 1:** Data-collection and refinement statistics for the BRIL–SRP2070Fab co-crystal Values in parentheses are for the highest resolution shell.

PDB code	7xrz
Data collection	
X-ray source	X10SA, SLS
Wavelength (Å)	1.00004
Temperature (K)	100.0
Detector	PILATUS 6M, Dectris
Crystal-to-detector distance (mm)	300.0
Total rotation range (°)	120.0
Rotation per image (°)	1.0
Exposure time per image (s)	1.0
Data processing	
Space group	*C*2
*a*, *b*, *c* (Å)	93.83, 75.24, 152.23
α, β, γ (°)	90, 99.67, 90
Resolution (Å)	48.64–2.10 (2.16–2.10)
*R*_merge_ (%)	6.3 (64.5)
No. of observations	134803 (10143)
No. of unique observations	57858 (4489)
Mean *I*/σ(*I*)	10.8 (1.5)
CC_1/2_	0.996 (0.568)
Completeness (%)	94.9 (94.7)
Multiplicity	2.3 (2.3)
Refinement statistics	
Resolution (Å)	42.74–2.10 (2.15–2.10)
No. of reflections	54939 (4064)
*R*_work_/*R*_free_ (%)	19.8/25.5 (31.0/32.3)
No. of atoms	
BRIL	1643
SRP2070Fab	6747
Water	665
Mean *B* value (Å^2^)	
BRIL	51.9
SRP2070Fab	39.3
Water	40.7
R.m.s.d.†	
Bond lengths (Å)	0.0041
Bond angles (°)	1.3104
Ramachandran plot	
Favored (%)	97.5
Allowed (%)	2.5
Disallowed (%)	0.0

† Root-mean-square deviation.
