# Supplementary material for: Structural insight into an anti-BRIL Fab as a G-protein-coupled receptor crystallization chaperone
Source: Acta Crystallogr D Struct Biol. 2023 Apr 26;79(Pt 5):435–41. doi: 10.1107/S205979832300311X (PMC10167669; doi:10.1107/S205979832300311X)
Supplement: Supplementary file 1 [file d-79-00435-sup1.pdf]

Acta Cryst D
STRUCTURAL BIOLOGY

Volume 79 (2023)

Supporting information for article:

Structural insight into an anti-BRIL Fab as a G-protein-coupled receptor crystallization chaperone

Hikaru Miyagi, Michihiko Suzuki, Mai Yasunaga, Hidetsugu Asada, So Iwata and Jun-ichi Saito

Table S1 Crystallization information

**Crystallization**	
Method	sitting drop vapor diffusion
Plate type	96 well
Temperature (°C)	4
Protein Concentration	~ 20 mg/mL
Buffer composition of protein solution	20 mM Tris-HCl (pH 7.6) and 2 mM 3-mercapto-1,2-propanediol
Composition of reservoir solution	18% (w/v) polyethylene glycol (PEG) 6000 and 100 mM Tris-HCl pH 8.5
Volume and ratio of drop	0.1 µL : 0.1 µL
Volume of reservoir	50 µL
Composition of the cryoprotectant	reservoir solution with 20% (v/v) glycerol
Drop setting	mosquito
Seeding	no

Table S2 Crystal packing information

(a) Interfaces of antigen-antibody and crystal packing (Top 10)

Molecule 1		Molecule 2		Symmetry operation	BSA (Å^2)	Type
Complex A	SRP2070Fab	Complex B	SRP2070Fab	(x, y, z)	1080.3	face-to-face
Complex B	SRP2070Fab	Complex B	BRIL	(x, y, z)	929.3	Ag-Ab
Complex A	SRP2070Fab	Complex A	BRIL	(x, y, z)	917.1	Ag-Ab
Complex B	SRP2070Fab	Complex B’	SRP2070Fab	(-x, y, -z+2)	799.9	back-to-back
Complex B	SRP2070Fab	Complex B’	BRIL	(x-1/2, y+1/2, z)	387.2	other packing
Complex A	SRP2070Fab	Complex A’	SRP2070Fab	(-x, y, -z+3)	373.7	back-to-back
Complex B	SRP2070Fab	Complex B’	SRP2070Fab	(x-1/2, y-1/2, z)	300.1	other packing
Complex A	BRIL	Complex A’	SRP2070Fab	(x-1/2, y-1/2, z)	292.3	other packing
Complex B	SRP2070Fab	Complex A’	SRP2070Fab	(x-1/2, y+1/2, z)	282.6	other packing
Complex A	SRP2070Fab	Complex A’	SRP2070Fab	(x-1/2, y+1/2, z)	218.0	other packing

Ag: antigen; Ab: antibody; Complex X’ is a symmetry mate of Complex X.

(b) Total surface area (SA) and BSA in crystal

Molecule		Total SA (Å^2)	Total BSA (Å^2)	Packing BSA (Å^2)*
Complex A	SRP2070Fab	19727.3	4409.0	3498.8
Complex A	BRIL	6124.4	1858.8	934.8
Complex B	SRP2070 Fab	20069.2	5431.6	4497.2
Complex B	BRIL	6040.9	1855.6	931.4

*Packing BSA = (Total BSA) - (BSA by antigen-antibody complex formation)

(c) Crystal packing interactions involved in the stacking of SRP2070Fab.

Complex / Chain	Residue No. [Atom]	Complex / Chain	Residue No. [Atom]	Interaction	Distance (Å)	packing
Complex A / L chain	Arg 155 [NH1]	Complex B / H chain	Glu 89 [OE2]	Salt bridge	2.7	face-to-face
Complex A / H chain	Glu 89 [OE1]	Complex B / L chain	Arg 188 [NE]	Salt bridge	3.2	face-to-face
Complex A / L chain	Asn 210 [ND2]	Complex B / H chain	Glu 62 [O]	H bond	3.2	face-to-face
Complex A / L chain	Asn 190 [ND2]	Complex B / H chain	Lys 63 [O]	H bond	2.9	face-to-face
Complex A / L chain	Asn 190 [ND2]	Complex B / H chain	Lys 65 [O]	H bond	3.1	face-to-face
Complex A / L chain	Asp 151 [O]	Complex B / H chain	Arg 40 [NH1]	H bond	2.6	face-to-face
Complex A / L chain	Asp 188 [O]	Complex B / H chain	Arg 67 [NZ]	H bond	3.9	face-to-face
Complex B / L chain	Arg 45 [NH2]	Complex B’/ L chain	Glu 81 [OE1]	Salt bridge	3.4	back-to-back
Complex A / L chain	Glu 79 [OE2]	Complex A’/ L chain	Ser 60 [OG]	H bond	2.7	back-to-back
Complex A / L chain	Glu 79 [OE1]	Complex A’/ L chain	Ser 60 [N]	H bond	2.9	back-to-back

Table S3 The interaction between SRP2070Fab and BRIL

(a) Interactions between SRP2070Fab H chain and BRIL

H chain CDR	Residue number [atom]	BRIL residue No._helix No. [atom]	Interaction	Distance (Å)
CDR1	Asp31 [OD2]	Lys83_IV [NZ]	Salt bridge	2..8
	Tyr33 [OH]	Asp74_III [OD2]	H bond	3.5
CDR2	Gly54 [O]	Lys77_III [NZ]	H bond	3.4
	Ser55 [OG]	Asp74_III [OD1]	H bond	2.5
	Asn57 [ND2]	Asp74_III [OD1]	H bond	2.9
CDR3	Ser101 [OG]	Glu92_IV [OE2]	H bond	2.4
	Tyr102 [N]	Glu92_IV [OE1]	H bond	2.6
	Tyr103 [OH]	Glu18_I [O]	H bond	3.6

(b) Interactions between SRP2070Fab L chain and BRIL

L chain CDR	Residue number	BRIL residue No._helix No.	Interaction	Distance (Å)
CDR1	Tyr32 [OH]	Lys95_IV [NZ]	H bond	2.4
CDR2	Tyr50 [OH]	Glu92_IV [OE1]	H bond	2.6
CDR3	Ser91 [O]	Thr96_IV [OG1]	H bond	3.1
	Asn92 [OD1]	Asn99_IV [ND2]	H bond	3.6
	Asn92 [O]	Asn99_IV [ND2]	H bond	3.0
	Trp94 [NE1]	Thr97_IV [OG1]	H bond	2.8

CDR: complementarity-determining region; H bond: hydrogen bond; H chain: heavy chain; L chain: light chain

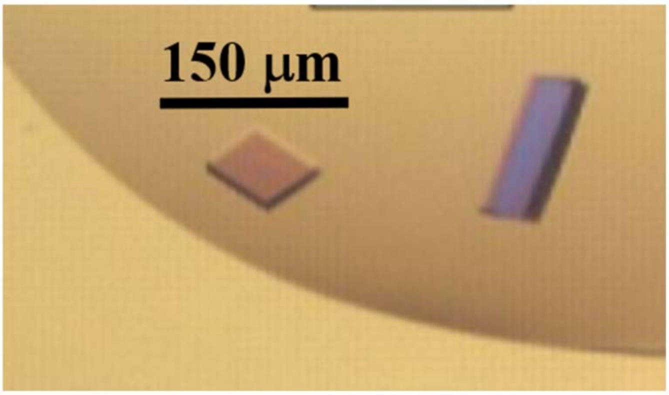

Figure S1 Crystals of BRIL/SRP2070Fab. The crystals of BRIL/SRP2070Fab were obtained under optimized conditions including 18% (w/v) PEG 6000 and 100 mM Tris-HCl pH 8.5 at 4°C.

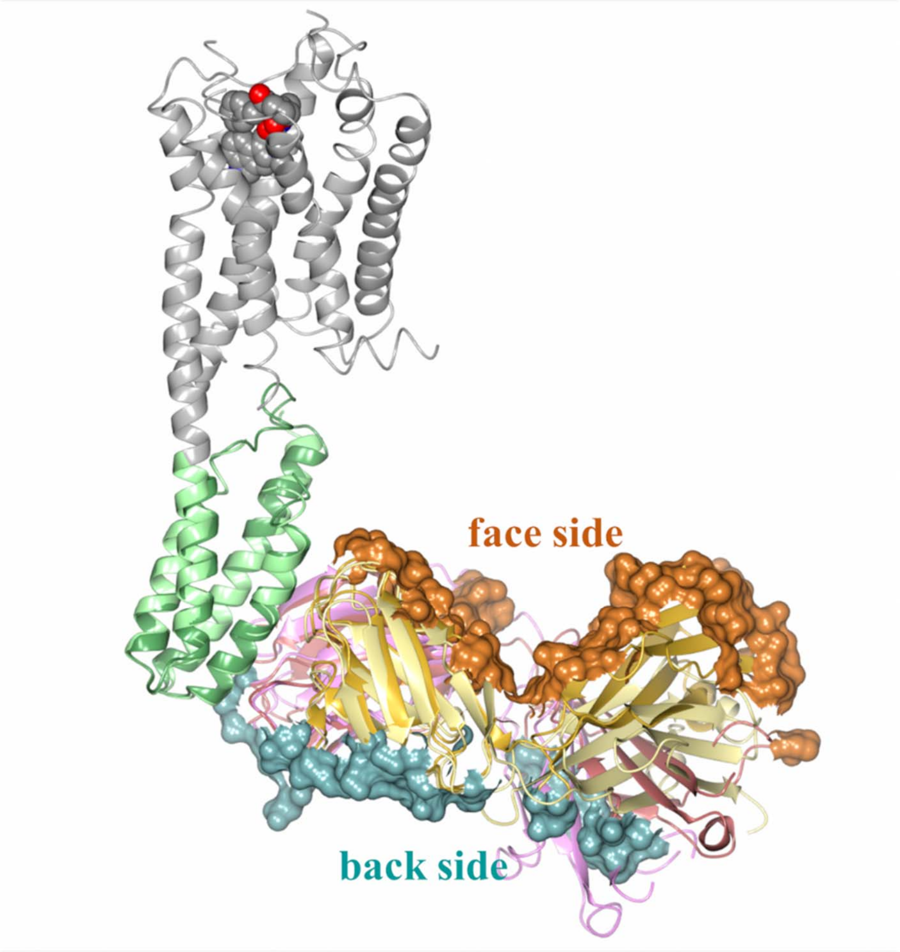

Figure S2 Definition of the face and back sides of SRP2070Fab. The BRIL/SRP2070Fab complex structure is superimposed on the $5HT_{1B}$-BRIL/SRP2070Fab complex (PDB ID: 7C61) on the BRIL portion. The main chains of the protein molecules are traced by ribbon models. BRIL, SRP2070Fab heavy chain, and SRP2070Fab light chain are indicated in green, dark red, and orange for the BRIL/SRP2070Fab complex and in light green, pink, and khaki for the 5HT1B-BRIL/SRP2070Fab complex, respectively. The receptor portion of the $5HT_{1B}$-BRIL/SRP2070Fab complex is grayed. The face and back sides of SRP2070Fab defined in this image are denoted by orange and dark cyan surfaces, respectively.

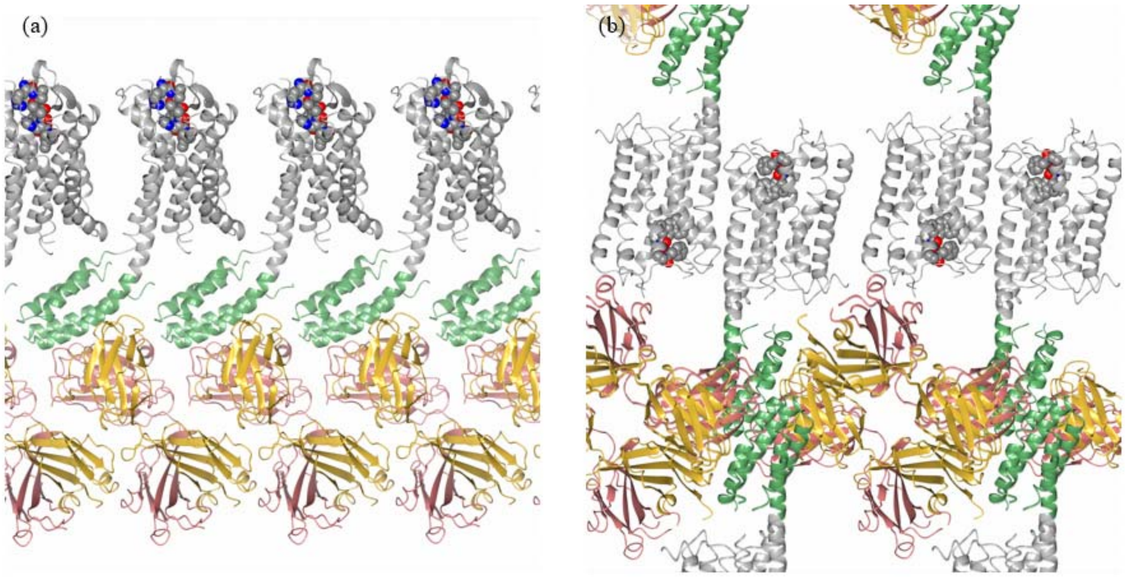

Figure S3 Stacking of SRP2070Fab molecules in G-protein–coupled receptor crystals. Crystal packing of (a) AT_2R-BRIL/SRP2070Fab (PDB ID: 7C6A) and (b) $5HT_{1B}$-BRIL/SRP2070Fab (PDB ID: 7C61) crystals. Protein structures are drawn as ribbon models. BRIL, SRP2070Fab heavy chain, and SRP2070Fab light chain are shown in green, dark red, and orange, while the receptor portions are grayed.

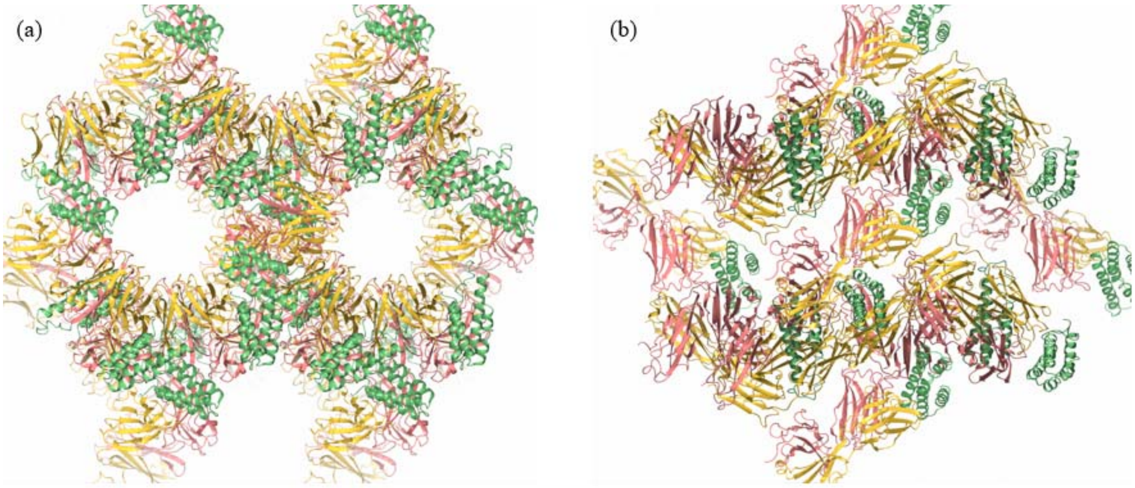

Figure S4 Molecular packing of the BRIL/BAG2 co-crystal. (a) Top and (b) side views of the contents of the BRIL/BAG2 co-crystal (PDB ID: 6CBV). Protein structures are drawn as ribbon models. BRIL, BAG2 heavy chain, and BAG2 light chain are shown in green, dark red, and orange, respectively.
